# Supplementary material for: A dark matter in sake brewing: Origin of microbes producing a Kimoto-style fermentation starter
Source: Front Microbiol. 2023 Feb 2;14:1112638. doi: 10.3389/fmicb.2023.1112638 (PMC9933502; doi:10.3389/fmicb.2023.1112638)
Supplement: Supplementary file 1 [file Data_Sheet_1.PDF]

frontiers

Supplementary Material

A dark matter in *sake* brewing: Origin of microbes producing a *Kimoto*-style fermentation starter

Kohei Ito[†*], Ryo Niwa[†], Ken Kobayashi, Tomoyuki Nakagawa, Genki Hoshino, Yuji Tsuchida

[†] **Equal contribution**

*** Correspondence:** Kohei Ito: kohei@biota.ne.jp

This file includes:

Supplementary figure 1-4

Supplementary Table 1

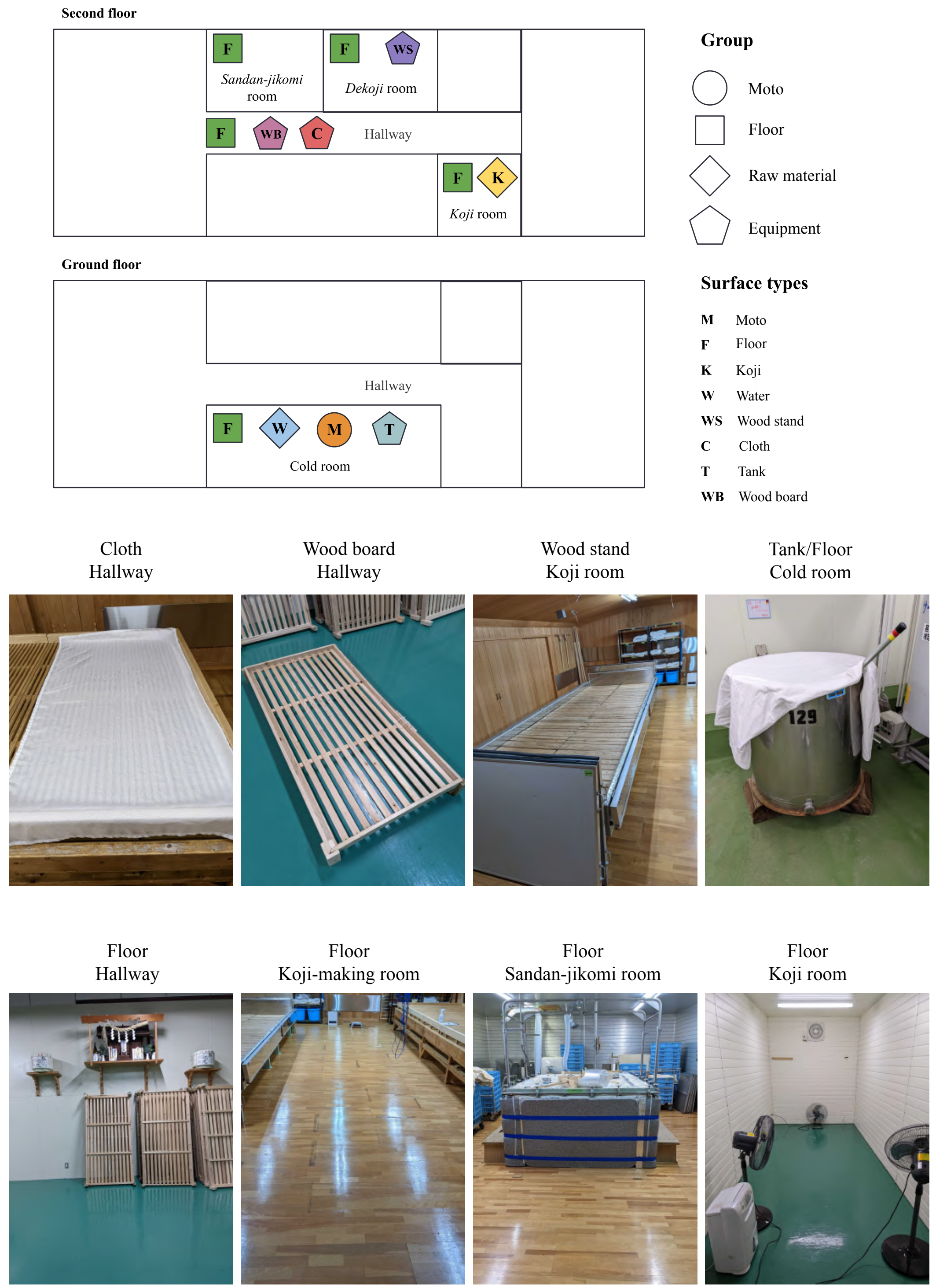

Supplementary Figure 1. A map of the brewery surveyed in this study. Shapes of the dots describe groups of samples. Pictures of equipment and floor are shown below the map.

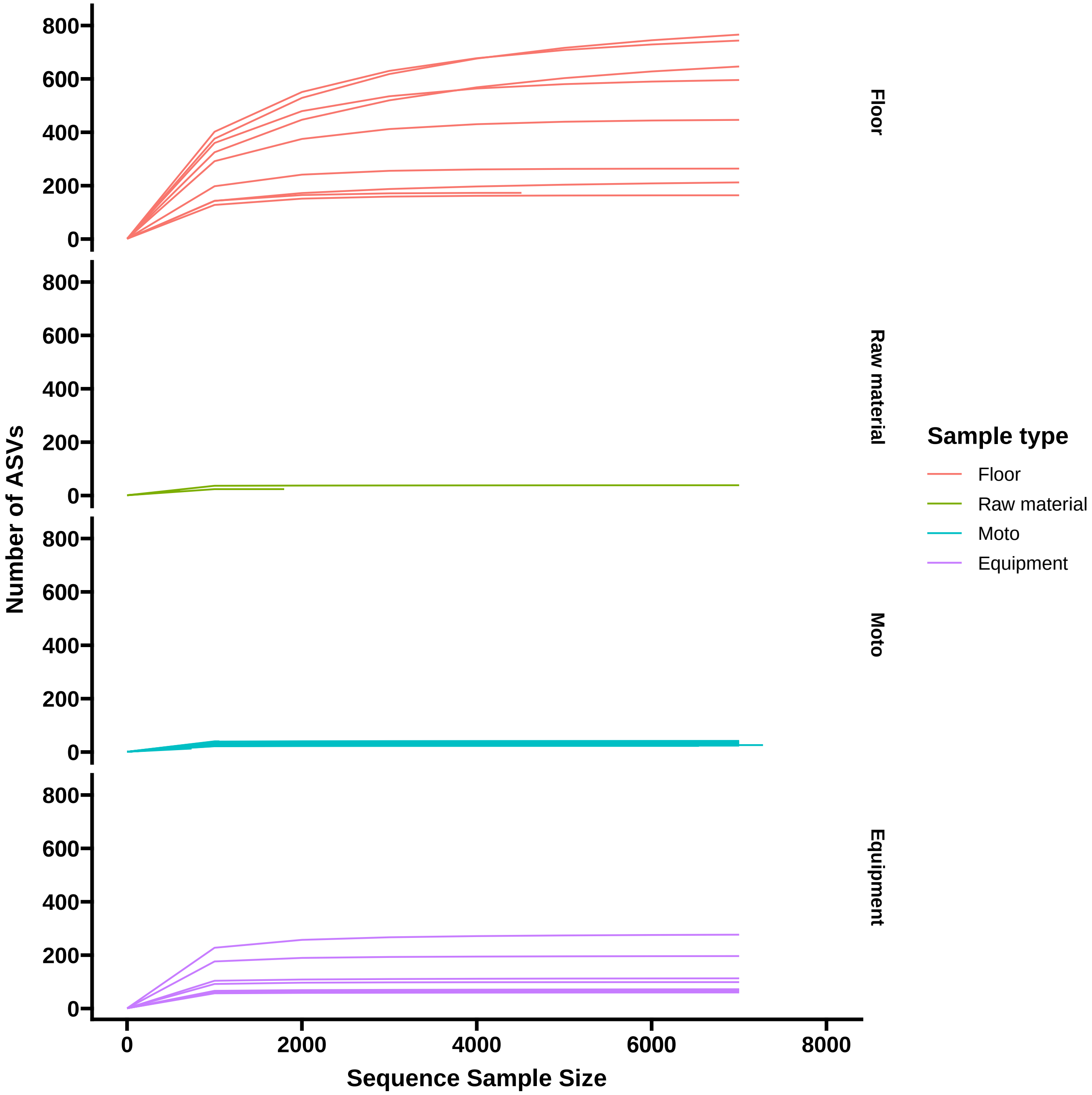

Supplementary Figure 2. A rarefaction curve showing the accumulation of the number of ASVs by sampling depth.

A

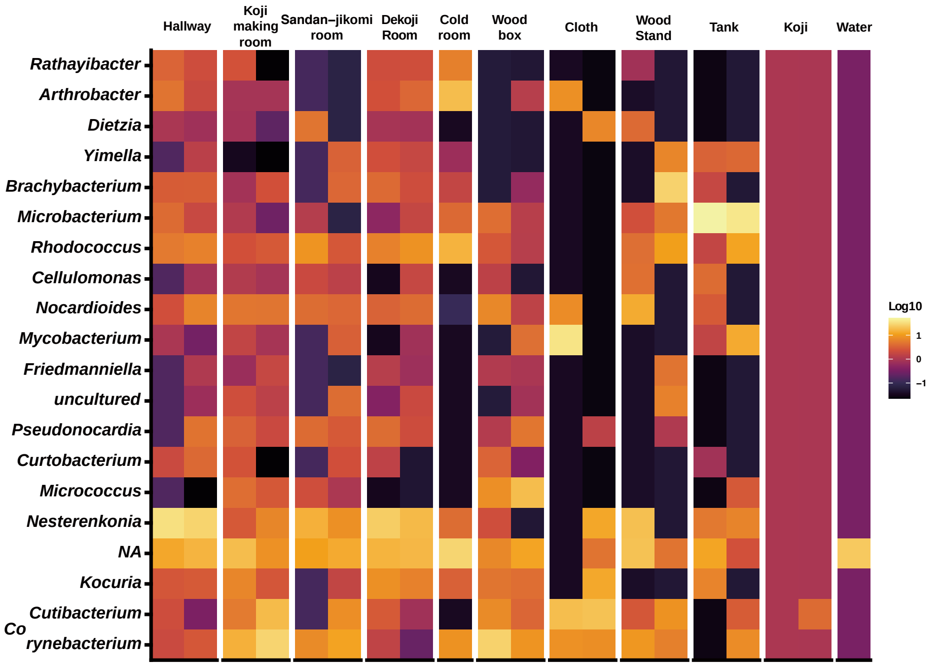
Hallway
Koji making room
Sandan–jikomi room
Dekoji Room
Cold room
Wood box
Cloth
Wood Stand
Tank
Koji
Water
Rathayibacter
Arthrobacter
Dietzia
Yimella
Brachybacterium
Microbacterium
Rhodococcus
Cellulomonas
Nocardioides
Mycobacterium
Friedmanniella
uncultured
Pseudonocardia
Curtobacterium
Micrococcus
Nesterenkonia
NA
Kocuria
Cutibacterium
Corynebacterium
Log10
1
0
−1

B

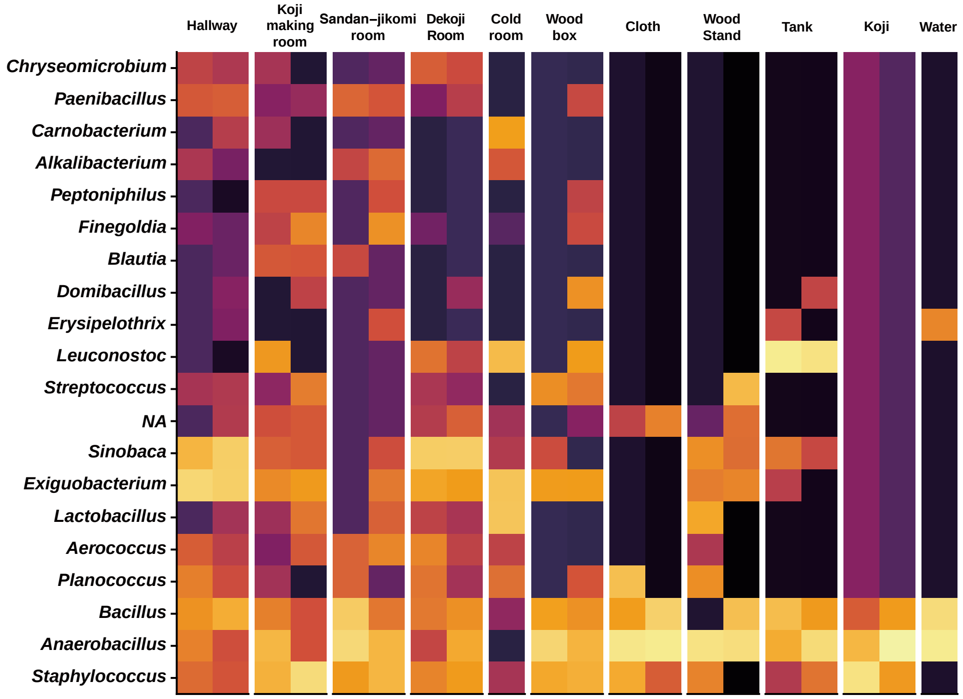
Hallway
Koji making room
Sandan–jikomi room
Dekoji Room
Cold room
Wood box
Cloth
Wood Stand
Tank
Koji
Water
Chryseomicrobium
Paenibacillus
Carnobacterium
Alkalibacterium
Peptoniphilus
Finegoldia
Blautia
Domibacillus
Erysipelothrix
Leuconostoc
Streptococcus
NA
Sinobaca
Exiguobacterium
Lactobacillus
Aerococcus
Planococcus
Bacillus
Anaerobacillus
Staphylococcus

C

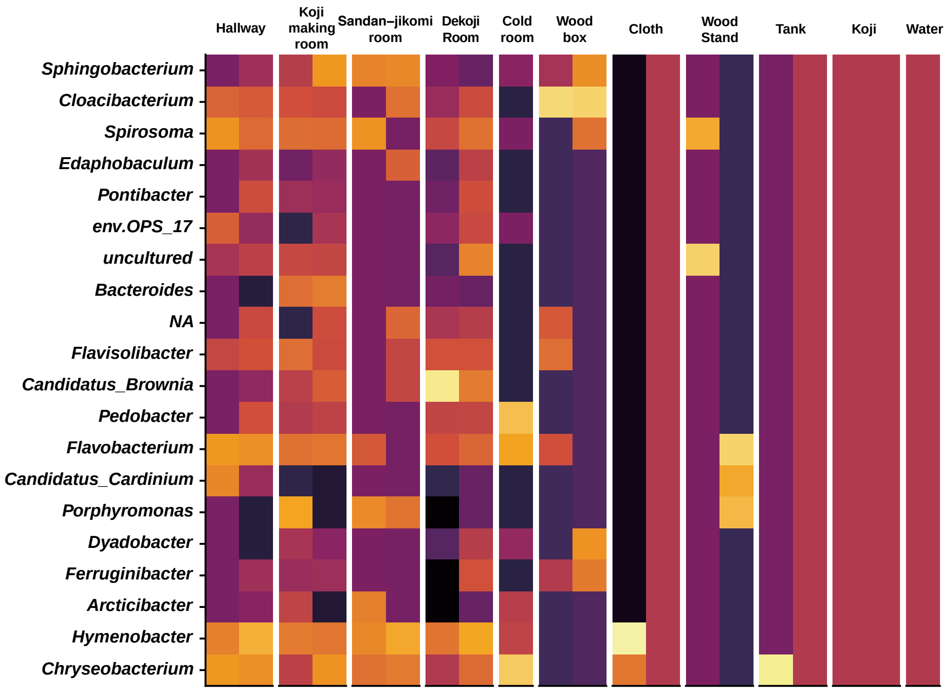

D

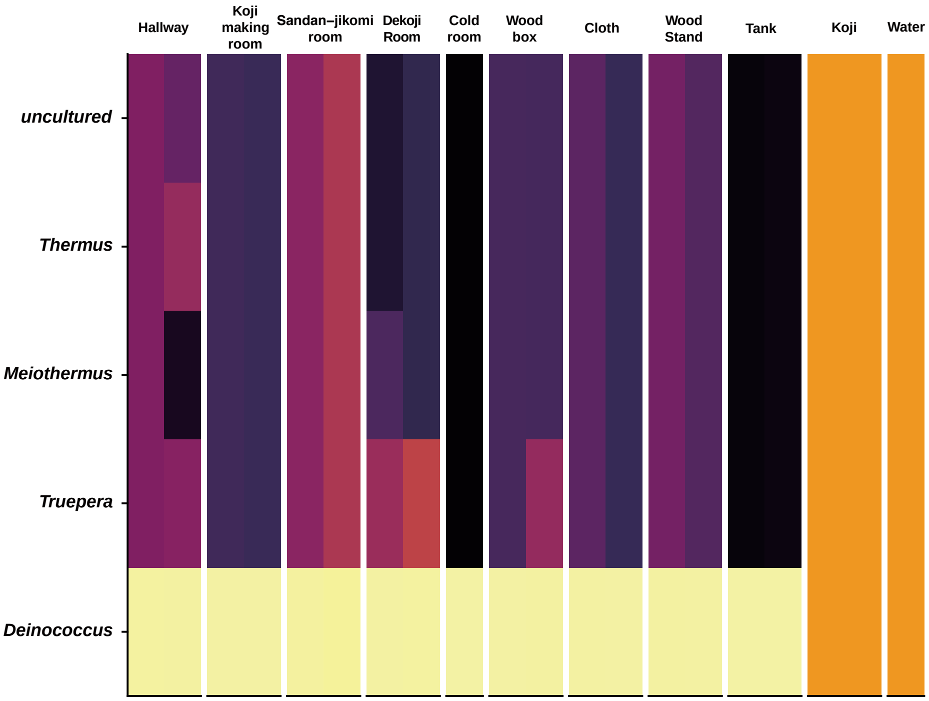

Supplementary Figure 3. Heatmaps showing the relative abundances (log10 scale) at the genus level of 4 phyla (*Firmicutes, Bacteroidota, Deinococcota, Actinobacteroidota*).

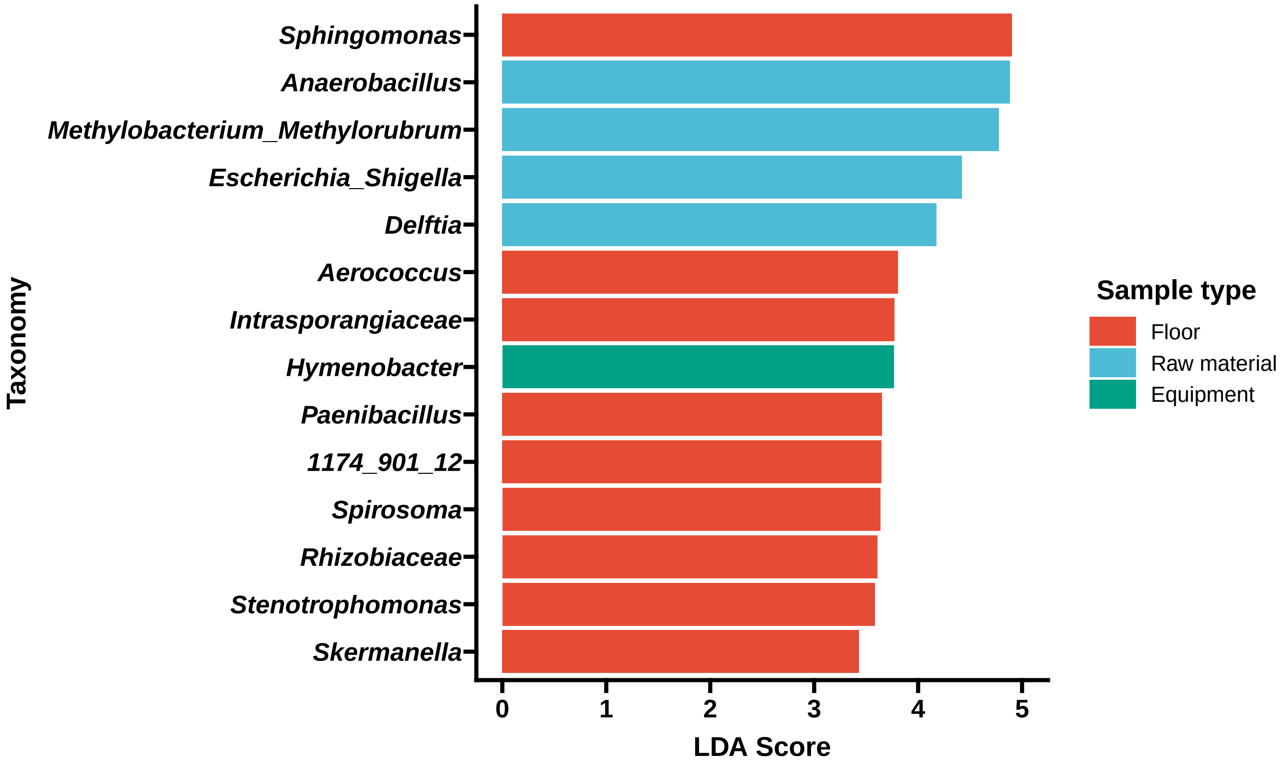

Supplementary Figure 4. Differential abundance microbes in the microbial communities by sample types from LEfSe computation.

Supplementary Table 1. A table of the number of reads per sample after the DADA2 denoising step.

sample-id	Sampling place	Reads
S01	cloth	27211
S02	cloth	21495
S03	wood stand	18052
S04	stand stand	21057
S05	wood board	13138
S06	wood board	15223
S07	tank	19977
S08	tank	19367
S09	water	17010
S10	koji	14918
S11	koji	17240
S12	Hallway	4724
S13	Hallway	27393
S14	Koji room	20794
S15	Koji room	29908
S16	Sandan-jikomi room	11027
S17	Sandan-jikomi room	9334
S18	Dekoji room	22469
S19	Dekoji room	11017
S20	Cold room	0
S21	Cold room	15978
